# Compartment Syndrome of the Flexor Compartment of the Arm Secondary to Pectoralis Major Tendon Rupture

**DOI:** 10.1155/2018/9042820

**Published:** 2018-12-13

**Authors:** M. A. Tranovich, J. B. Stirton, J. C. Maier, M. B. Tanios, J. E. Lea, N. A. Ebraheim, J. D. Miller

**Affiliations:** ^1^Department of Orthopedics, University of Toledo Medical Center, MS 1094, 3000 Arlington Ave., Toledo, OH 43614, USA; ^2^University of Toledo School of Medicine, 3000 Arlington Ave., Toledo, OH 43614, USA

## Abstract

*Case*. Compartment syndrome following muscle rupture is a rare entity with few mentions in the literature. We present a case of pectoralis major rupture in a 38-year-old male that evolved into compartment syndrome of the anterior compartment of the arm. Rupture of the pectoralis is uncommon and most often occurs during weight lifting. Compartment syndrome secondary to this injury is extremely uncommon, with only one reported case in the pectoralis major itself and several cases of biceps compartment syndrome. Due to the potentially devastating consequences of a missed compartment syndrome, it is imperative that physicians maintain a high level of suspicion in patients with these unusual injuries presenting with severe swelling and pain.

## 1. Introduction

Acute compartment syndrome is an orthopedic emergency that develops when the pressure in a muscle compartment reaches a level that compresses neurovascular structures and compromises blood supply. As muscles swell after acute injury, the dense fascia that encases the muscle and associated vessels and nerves does not expand to accommodate for the increase in volume, leading to an increase in pressure. When the intracompartmental pressure exceeds 30–40 mmHg or when the pressure is within 30 mmHg of the diastolic blood pressure, an immediate fasciotomy is indicated [1]. Prolonged hypoperfusion leads to irreversible tissue death which in turn may lead to significant functional impairment, loss of limb, and in, rare cases, death [2]. Compartment syndrome occurs most commonly in the leg and forearm, and 2/3 of cases are associated with fractures. The highest incidence has been observed in patients in their second and third decades. It has been postulated that younger patients have increased muscle bulk and thus less room in their compartments to allow for swelling [3].

Compartment syndrome secondary to pectoralis major injury is a very rare phenomenon. A literature search yielded only two cases of compartment syndrome secondary to pectoralis major trauma. Tarkin et al. reported a case of exercise-induced compartment syndrome of the pectoralis major and other shoulder adductors in a tree climber, while Smith et al. presented a case of compartment syndrome of the biceps secondary to a pectoralis major tendon torn while bench pressing [4, 5]. It is generally agreed that pectoralis major tears are infrequent injuries. However, researchers also believe the incidence is underreported [6]. Schepsis et al. found that weightlifting—more specifically, bench pressing—is the most common cause of pectoralis trauma [7].

Compartment syndrome secondary to biceps brachii injury has also been recognized as an uncommon occurrence. Three cases of compartment syndrome of the anterior arm have been reported following rupture of the long head of the biceps [8–10]. Of note, all three patients were elderly males on long-term anticoagulation with Coumadin. Lanier et al. reported an additional case of compartment syndrome of the anterior arm following distal biceps rupture in an otherwise healthy 33-year-old male, while Grandizio et al. reported the same in a healthy 45-year-old male after distal biceps rupture [11, 12]. The lower rate of compartment syndrome in the upper arm as compared to the forearm or leg has typically been attributed to the relatively thinner and more distensible brachial fascia [13].

We present the case of a 38-year-old male with compartment syndrome of the flexor compartment of the arm secondary to a pectoralis major tendon rupture suffered while bench pressing. Informed consent was obtained from the patient to share his case and images.

## 2. Case

**Figure 1:**
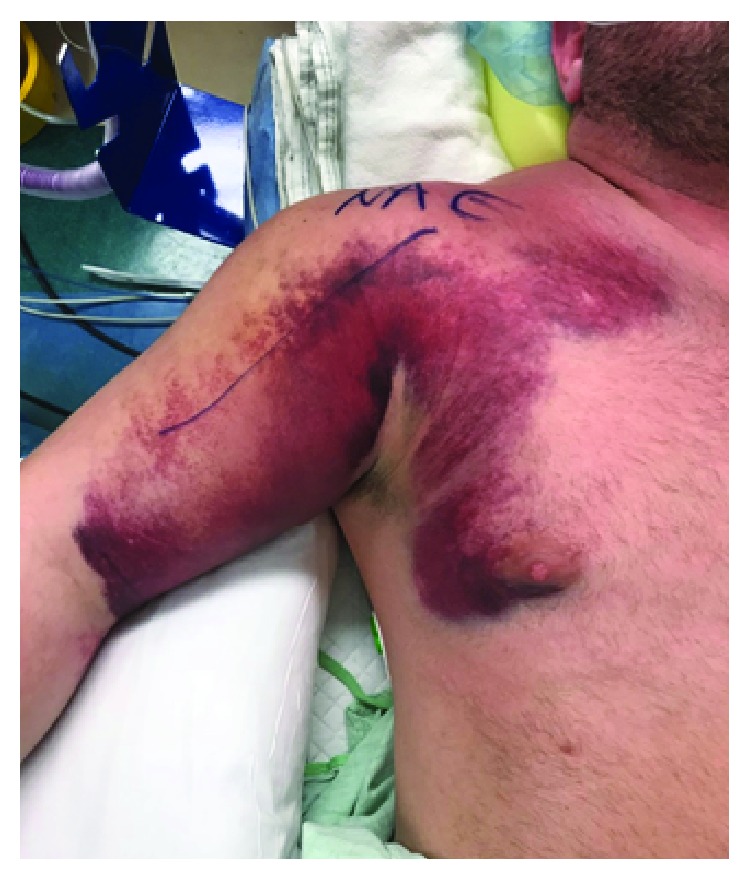
Deformity of anterior axillary fold.

**Figure 2:**
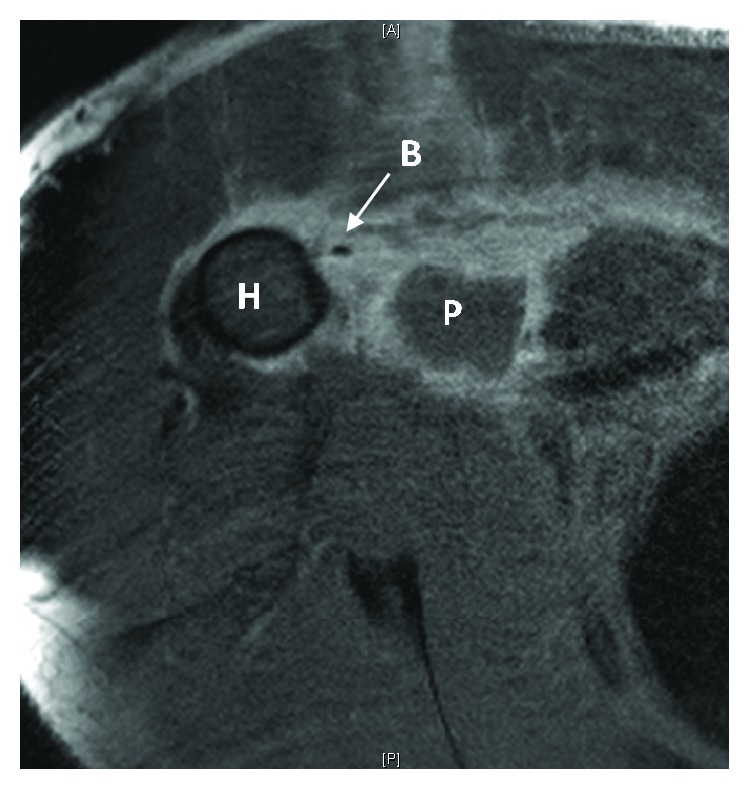
Axial MRI image. H: humerus; P: pectoralis major; B: biceps tendon.

**Figure 3:**
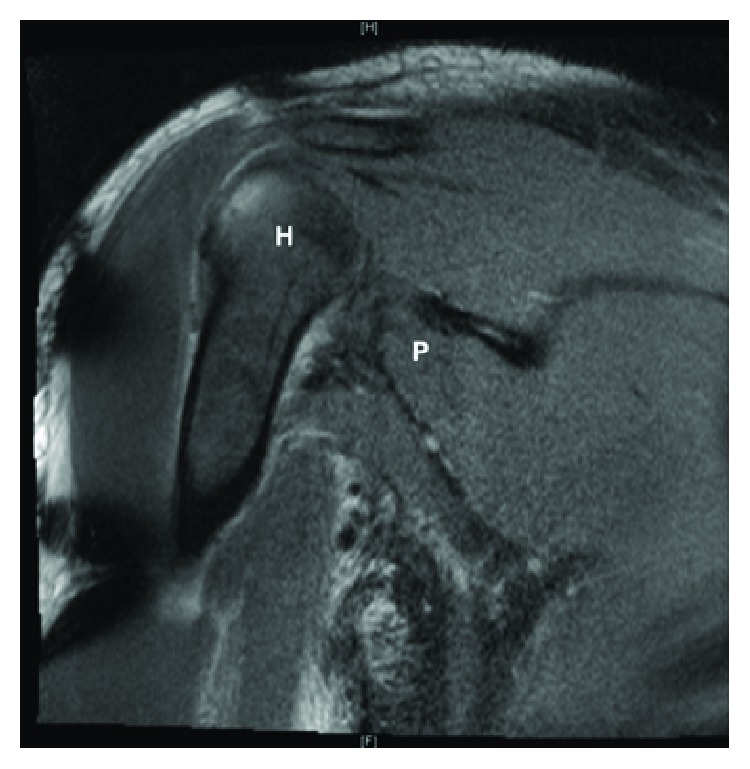
Coronal MRI image. H: humerus; P: retracted pectoralis major.

**Figure 4:**
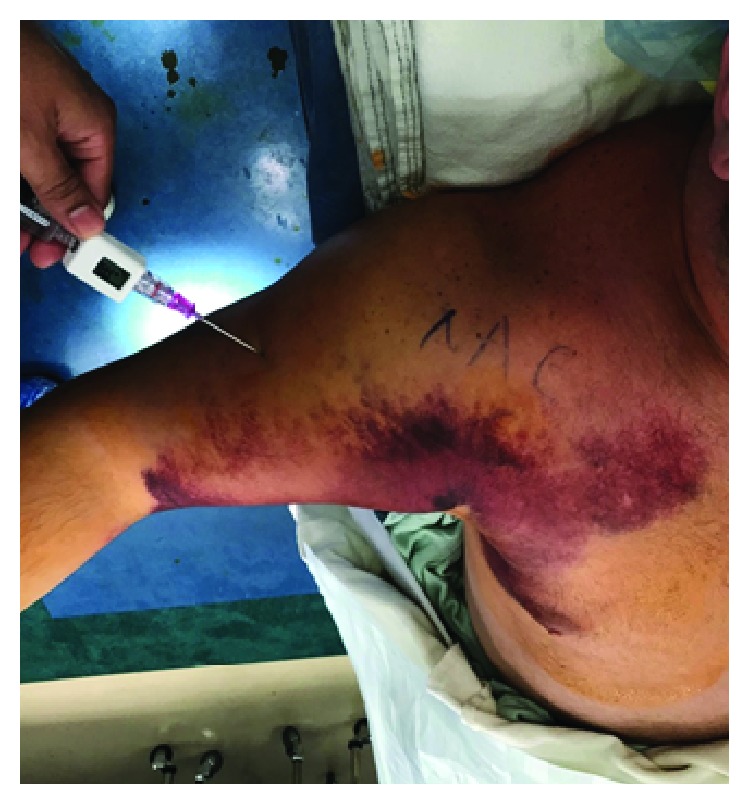
Anterior arm compartment pressure checks preoperatively: 37, 39, and 42 mmHg.

Our patient is a 38-year-old male who presented with right chest wall and shoulder pain after a weight lifting injury. The patient was performing a one-rep max bench press when he felt a pop in his right upper arm, accompanied by severe pain. There was no history of anabolic steroid use. He was initially treated with ice and a sling by a trainer and presented to the emergency department for further evaluation. Plain films were negative for fracture or dislocation and the patient was neurovascularly intact, so he was discharged home by ER staff in the sling. He presented to the orthopedic clinic the following day with moderate pain in the chest and arm. He denied numbness and paresthesia. On physical examination, there was a large amount of swelling and ecchymoses throughout the right upper arm extending into the pectoralis major muscle belly. Additionally, there was a large bulge in the anterior chest with loss of contour of the axillary fold (Figure 1). The patient had full active range of motion of the elbow, wrist, and digits. He was sensory intact throughout the right upper extremity with a 2+ radial pulse. An MRI was scheduled to determine the extent of the injury and to aid in surgical planning. The patient was given oxycodone and valium to alleviate the pain and muscle spasms until surgery, which was scheduled after his MRI. The MRI demonstrated avulsion of the pectoralis major tendon from its insertion on the humerus with retraction as well as strain of the anterior deltoid (Figures 2 and 3). He was scheduled for surgery in five days. Two days later, the patient returned to our facility with severe worsening pain in the right upper arm. Intracompartmental pressure readings in the anterior compartment of the arm taken about the midpoint of the biceps at the point of maximal swelling were 37, 39, and 42 mmHg with a diastolic blood pressure of 71 mmHg (Figure 4). Thus, with a diagnosis of compartment syndrome confirmed, we proceeded to the operating room for an emergency fasciotomy with repair of the pectoralis major tendon rupture.

**Figure 5:**
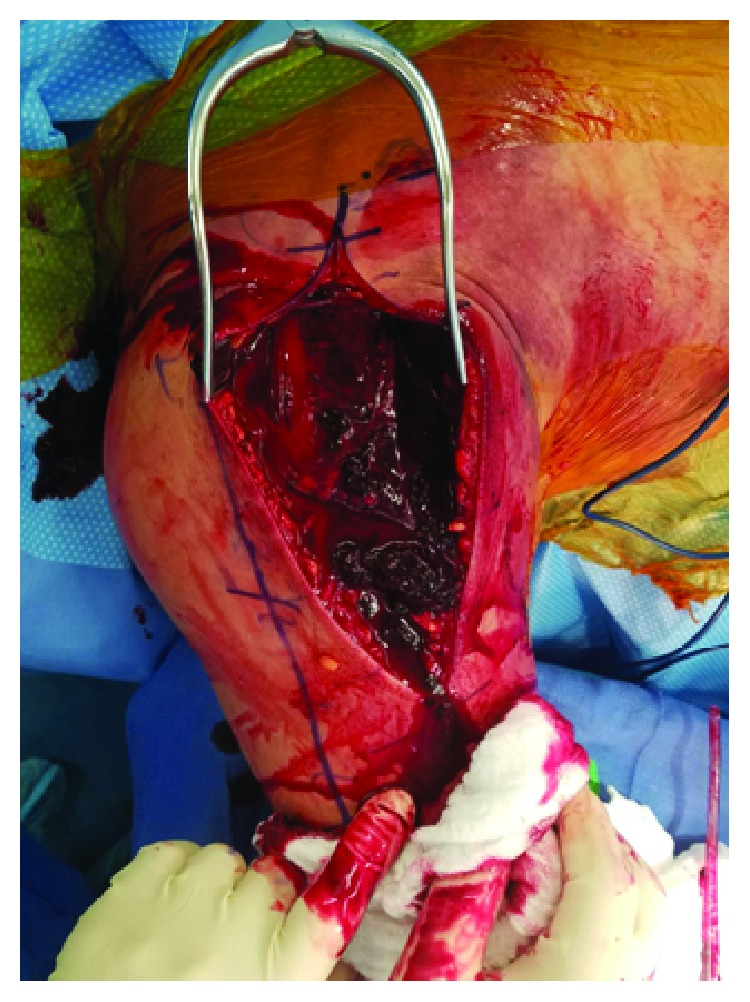
Muscle bulge and clot extravasation with compartment release.

**Figure 6:**
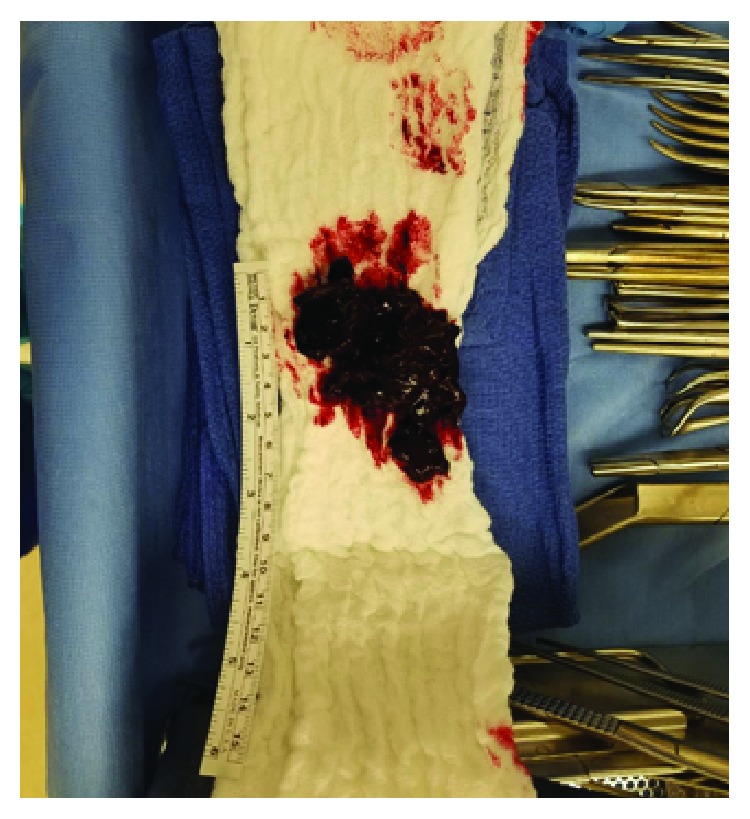
Hematoma evacuated from the anterior compartment of the arm.

**Figure 7:**
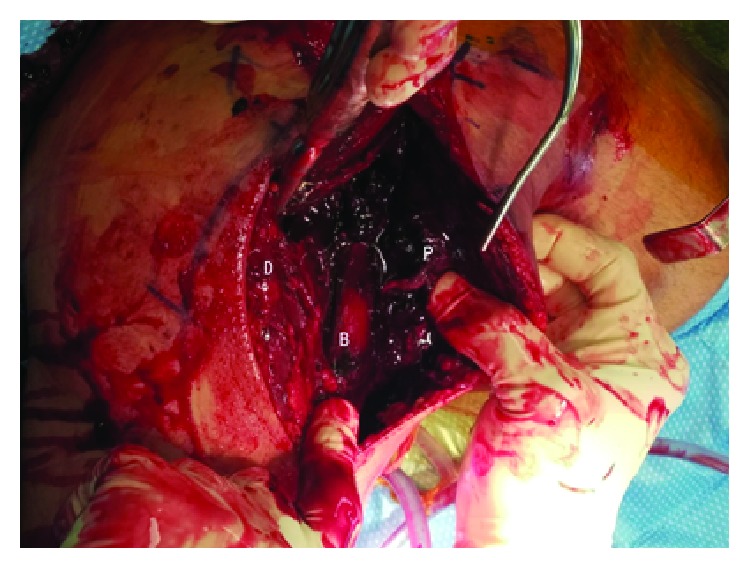
D: deltoid; P: ruptured pectoralis major tendon; B: biceps brachii.

**Figure 8:**
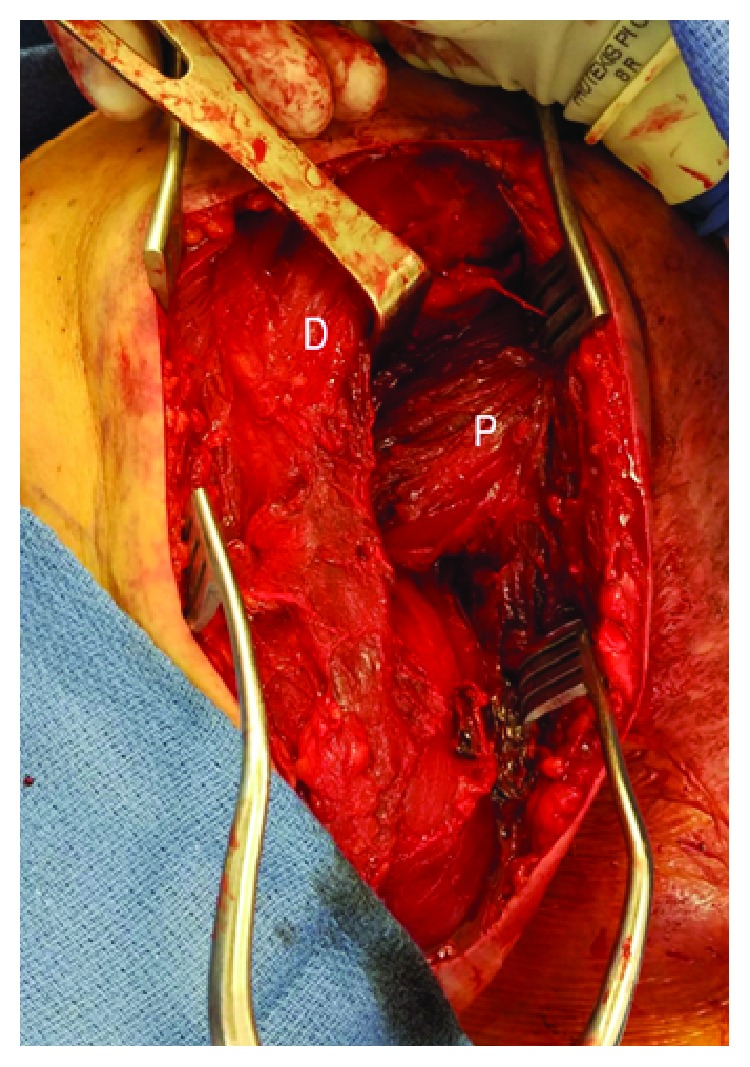
D: deltoid; P: repaired pectoralis major.

An extended deltopectoral approach was used, and the deltopectoral and biceps fascia were released. Immediately, a large amount of hematoma was expelled and the muscle bellies visibly bulged from the incision sites (Figures 5 and 6). All muscles still appeared viable. No apparent vascular damage was noted. Upon further dissection, both heads of the pectoralis major were found to be avulsed from the proximal humerus (Figure 7). After preparation of the footprint with curette and rongeur, three double-loaded 4.5 mm Mitek suture anchors (DePuy Synthes, Raynham, MA) were placed lateral to the bicipital groove for the repair of the tendon. The proximal and distal suture anchors were used such that one suture of each was run in a Krakow fashion along the superior and inferior aspects of the tendon, respectively. The remaining suture from each of those anchors was passed in a horizontal mattress fashion medial to the Krakow stitches. The middle suture anchor was used to place a horizontal mattress stitch with a medial ripstop stitch (Figure 8). The wound was irrigated, and a negative pressure dressing was applied. The patient was made nonweightbearing and placed in a sling with a circumferential strap to ensure adduction of the arm. The patient returned to the operating room four days later to undergo irrigation and debridement with a tension-free primary wound closure. He was again placed into his sling and given strict instructions to avoid abduction and external rotation of the arm. The patient did well postoperatively and was discharged home in a stable condition that same day with a one-week follow-up appointment. He continued to do well and was instructed to remain nonweightbearing in his sling for a total of 6 weeks before beginning formal therapy. Gentle stretching and passive range of motion were then begun, followed by strengthening exercises at the 12th week mark. At his four-month follow-up, the patient had active forward flexion of the shoulder to 150°, abduction to 150°, and external rotation of 50°. His rotator cuff, biceps, triceps, wrist extensors, wrist flexors, and interossei all demonstrated 5/5 strength. There were no sensory deficits on examination. He continues to attend therapy for motion and strengthening and has a lifting restriction of <5 pounds at work.

## 3. Discussion

Although compartment syndrome is most often seen following high-energy trauma, there are a number of case reports of compartment syndrome in both the upper and lower extremities related to weightlifting. In 2014, Bunting and Briggs described a case of acute exertional compartment syndrome in the bilateral supraspinatus muscles of a 23-year-old male the day after an increase in weightlifting activity. MRI demonstrated myositis and diffuse edema in the supraspinatus bilaterally, and the patient was taken to the operating room for emergency fasciotomies, which completely relieved his pain [14]. Segan et al. reported a case of compartment syndrome in the bilateral biceps of an experienced weight lifter necessitating extended fasciotomies of the anterior arm and forearm [15]. Additionally, a case of compartment syndrome in the thigh of a 16-year-old male 48 hours after performing quadriceps extensions with heavy weights has been described. The patient successfully underwent fasciotomies by a single lateral incision [16].

To our knowledge, compartment syndrome of the anterior arm related to the rupture of the pectoralis major has only been reported once in the literature. Our patient had delayed onset of compartment syndrome of the anterior compartment of the arm three days after rupturing his pectoralis major tendon while bench pressing. Although he initially was stable after his injury, upon second presentation, our patient had a significant increase in both pain and swelling of the anterior chest and arm. The clinical picture was similar to that reported by Smith et al. [5]. Fortunately, the patient's clinical diagnosis was confirmed in a timely manner with compartment pressure checks and he was taken emergently to the operating room. The large hematoma expelled upon incision indicated the patient's compartment syndrome to be secondary to infiltration of the anterior arm following his traumatic tendon injury. Surgeons must keep in mind that compartment syndrome may be associated with any number of soft tissue or bony injuries, and any patient presenting with clinical signs and symptoms should be diagnosed and treated appropriately with emergent fasciotomies.
